# Efficacy of *Phlomis crinita* Extract-Loaded Nanostructured Formulation in Accelerating Wound Healing

**DOI:** 10.3390/pharmaceutics17091093

**Published:** 2025-08-22

**Authors:** Tahsine Kosksi, Paola Bustos-Salgado, Arem Selmi, Marwa Rejeb, Nawres Debbabi, Lupe Carolina Espinoza, Lilian Sosa, Joaquim Suñer-Carbó, Mohamed Ali Lassoued, Leila Chekir-Ghedira, Ana Cristina Calpena

**Affiliations:** 1 Laboratory of Natural Bioactive Substances and Biotechnology (LR24ES14), Faculty of Dental Medicine, University of Monastir, Av. Avicenne, Monastir 5019, Tunisia; tahsine223@gmail.com (T.K.);; 2 Departament de Farmàcia i Tecnologia Farmacèutica, i Fisicoquímica, Facultat de Farmàcia i Ciències de l’Alimentació, Universitat de Barcelona (UB), Av. Joan XXIII, 27-31, 08028 Barcelona, Spain; 3 Departamento de Química, Facultad de Ciencias Exactas y Naturales, Universidad Técnica Particular de Loja, San Cayetano Alto, Loja 1101608, Ecuador; 4 Institut de Nanociència i Nanotecnologia, Universitat de Barcelona (UB), Av. Diagonal 645, 08028 Barcelona, Spain; 5 Institute of Microbiology Research (IIM), Faculty of Sciences, National Autonomous University of Honduras (UNAH), Tegucigalpa 11101, Honduras; 6 Pharmaceutical Technology Research Group, Faculty of Chemical Sciences and Pharmacy, National Autonomous University of Honduras (UNAH), Tegucigalpa 11101, Honduras; 7 Laboratory of Pharmaceutical, Chemical and Pharmacological Drug Development (LR12ES09), Faculty of Pharmacy of Monastir, University of Monastir, Monastir 5000, Tunisia

**Keywords:** nanostructured formulation, *Phlomis crinita*, wound healing, drug delivery, skin integrity, nanoemulsion

## Abstract

**Background/Objectives**: Recent advancements in innovative drug delivery nanosystems have significantly impacted wound healing, particularly through the incorporation of natural products. This study aimed to develop and characterize a *Phlomis crinita* extract-loaded nanostructured formulation (PCE-NF) as a topical therapy for skin wounds. **Methods**: This study involved the incorporation of *P. crinita* extract in a nanoemulsion by the high-energy emulsification method. This formulation was subjected to physicochemical and biopharmaceutical characterization, and a physical stability study over 30 days. Biocompatibility, tolerability, and irritant effects were assessed, while the wound healing potential was evaluated using in vitro skin models of fibroblasts and keratinocytes. **Results**: PCE-NF showed a homogeneous appearance with nanometric-sized spherical droplets of 212.27 nm and Newtonian behavior. This formulation showed a sustained release of its majority component (luteonin 7-(6″-acetylglucoside)), which followed a hyperbolic kinetic while showing high permeation, through healthy human skin, with 22.01 µg after 27 h. There were no cytotoxic effects of PCE-NF with improvements in skin barrier function and hydration levels. The wound healing potential of PCE-NF at 3.125 µg/mL was evidenced by enhanced cell migration and accelerated wound closure in 3T3-L1 and HaCaT cells, with values of 94.24 and 92.41%, respectively. **Conclusions**: These results suggest that this formulation could be used as an effective wound healing treatment.

## 1. Introduction

Wound healing is a vital biological process that involves various phases, such as hemostasis, inflammation, proliferation, and remodeling [1]. Re-epithelialization, which primarily involves the restoration of the epithelium by keratinocytes during the proliferative phase, is an essential aspect of wound healing. This process is highlighted by several key activities, including the formation of new blood vessels and the proliferation and migration of various cells, particularly keratinocytes and fibroblasts [2]. Following skin injury, inflammatory cells are drawn to the injured site to eliminate damaged tissues. Afterwards, the activated keratinocytes begin to migrate from the wound edge toward the denuded area where they proliferate to cover the exposed area and facilitate closure [3]. Meanwhile, fibroblasts, which also play a crucial role in the recovery of the wounded dermis, begin to multiply, spread, and migrate into the wound region, producing a new extracellular matrix (ECM) and expressing dense actin bundles [4]. Thus, keratinocytes and fibroblasts cooperate to repair the integrity and functionality of the skin, making them essential for efficient wound healing.

Proper management of wounds is crucial for preventing severe infection, accelerating wound healing, reducing scars and pain, and facilitating recovery. However, the traditional treatments available often face challenges, including a low bioavailability of active ingredients and insufficient penetration through the skin barrier. The outer layer of the skin, known as the *stratum corneum*, acts as a crucial barrier restricting the entry of drugs into deeper skin layers [5]. Most drugs applied topically face difficulties penetrating this layer due to its protective function. This limitation can reduce the efficacy of drug treatments, as they may not reach the intended target sites within the skin, or those sites may not receive sufficient drug exposure [6]. To overcome this barrier and enhance drug delivery, various strategies are employed to modify the skin penetration profiles of drugs, including the use of penetration enhancers, physical methods, and nanotechnology-based drug delivery systems [7].

Natural substances offer effective therapeutic options, have fewer side effects, and are typically more affordable. In fact, various studies have shown the effectiveness of plant-based products in the healing of wounds and burns, in which they help accelerate the healing process [8,9,10]. *Phlomis crinita*, a Lamiaceae species prevalent in North Africa, is known traditionally for its use in the treatment of wounds and burns [11]. Previous studies have investigated the pharmacological properties of this plant, including its free radical scavenging potential, as well as its antioxidant, anti-inflammatory, antigenotoxic, antibacterial, and wound healing properties [11,12,13]. Numerous compounds have been identified for this plant, including luteonin 7-(6″-acetylglucoside) “luteolin-acetylglucoside”, a natural flavonoid that has shown healing properties in both in vitro and in vivo studies. These studies have suggested that this compound promotes the proliferation and migration of fibroblasts and increases the expression of E-cadherin, which improves the generation of stem cells promoting wound healing and reducing scarring and inflammation [13,14,15,16].

In addition to the benefits of medicinal plants for wound care, an innovative approach is to integrate them into nanosystems. This approach not only improves bioavailability but also enhances the storage stability of these compounds, preventing their degradation [17]. It also allows for targeted delivery and the sustained and controlled release of bioactive compounds, effectively directing them to specific sites of action while helping to overcome the skin barrier [18,19]. Nanoemulsions are colloidal systems formed by nanometer-sized droplets of one liquid dispersed in another liquid that provide an effective method for delivering active ingredients to the skin, thanks to their thermodynamic stability, large surface area, and ability to penetrate the skin, improving the bioavailability of poorly soluble drugs and facilitating their absorption [20,21].

Based on this context, the aim of this study was to develop and characterize a topical nanoemulsion formulation incorporating *Phlomis crinita* extract (PCE-NF), and to evaluate its efficacy in promoting the migration and proliferation of keratinocytes and fibroblasts—key cellular mediators in the wound healing process. Given that luteolin-acetylglucoside is a principal bioactive constituent of the extract, it was selected as a marker compound for the standardization of the formulation.

## 2. Materials and Methods

### 2.1. Materials

Caprylocaproyl polyoxyl-8-glyceride (Labrasol^®^), medium chain triglycerides (Labrafac^®^ lipophile WL 1349), and Polyglyceryl-3-dioleate (Plurol^®^ oleique CC 497) were gifted by Gattefosse (Gennevilliers, France). Propylene glycol was purchased from Sigma-Aldrich (Madrid, Spain). The fetal bovine serum, DMEM medium, trypsin, HEPES, streptomycin, and penicillin used in this study were acquired from Sigma Cell Culture (Courtaboeuf, France). The dialysis membrane (12 kDa, Dialysis Tubing Visking) was obtained from Medicell International Ltd. (London, UK). Thiazolyl blue tetrazolium bromide (MTT), a membrane-permeable dye, was obtained from Abcam (Paris, France). A Millipore Milli-Q water purification system (Millipore Corporation; Burlington, MA, USA) was used.

### 2.2. Extract Preparation

*Phlomis crinita* leaves were collected in the region of Jammel, Monastir (Tunisia), from wild-growing plants. This species was identified based on the morphological criteria described in the Flore de la Tunisie [22]. A voucher specimen (PC-11.06) has been deposited at the Herbarium of the Faculty of Pharmacy, University of Monastir, Tunisia. The powdered leaves of *P. crinita* were macerated in a hydroethanolic mixture (80:20) for 72 h and filtered, and the ethanol was evaporated under low pressure at 40 °C. The obtained extract was lyophilized and stored until further use.

### 2.3. Quantification of the Luteolin-Acetylglucoside from Phlomis crinita Extract

The amount of luteolin-acetylglucoside in the *Phlomis crinita* extract was quantified using a previously validated high-performance liquid chromatography (HPLC) method described in our previous work [14]. Briefly, the assay was performed using a Waters^®^ 2695 separation module (Milford, MA, USA) equipped with a diode-array detector, and an Atlantis^®^ C18 column (250 mm × 4.6 mm, 5 µm). The mobile phase consisted of acidified water (5% glacial acetic acid) and acetonitrile under isocratic elution with a flow rate of 0.6 mL/min. The detection wavelength was set at 330 nm, and the injection volume was 50 µL. The calibration curve obtained was from 6.25 to 200 µg/mL of luteolin-acetylglucoside. The data analysis was performed using Empower 3^®^ software (V.7.3.2). The limit of detection (LOD) and limit of quantification (LOQ) were confirmed to be 3.47 ± 1.39 µg/mL and 10.52 ± 4.21 µg/mL. Selectivity was further confirmed by comparison of chromatograms from the pure standard, the final nanoemulsion, and the blank formulation (without extract). No interfering peaks were detected at the analyte retention time in the blank sample, and both the standard and formulation samples showed a single peak at 6.57 min (Figure S1, Supplementary Material).

### 2.4. Preparation of Nanostructured Formulations

A *Phlomis crinita* extract-loaded nanoemulsion (PCE-NF) was prepared using the high-energy emulsification method via the ultrasonic homogenization process [23]. Several compositions were screened by varying the relative concentrations of Labrafac^®^ (oil phase), Labrasol^®^ (surfactant), and Plurol^®^ Oleique (co-surfactant) in order to optimize extract solubilization and physical stability. These mixtures were sonicated for 15 min in an Elma Transonic Digital S T490 DH ultrasonic bath (Elma, Singen, Germany) and then stirred in a water bath at 32 °C for 15 min. The final composition was selected based on the formulation in which the *P. crinita* extract was fully solubilized and no phase separation occurred after centrifugation (4000 rpm, 30 min) or freeze–thaw stress (three cycles). These criteria ensured the selection of a homogeneous and robust formulation suitable for topical administration. Blank-NF was also prepared using the same method without PC extract.

### 2.5. Physicochemical Characterization

The droplet size (Z-ave) and polydispersity index (PdI) of PCE-NF were measured using Zetasizer Nano ZS (Malvern Instruments, Malvern, UK). The measurements were conducted in triplicate at 25 °C.

The pH determination of the nanoformulation was performed in triplicate (*n* = 3), at room temperature, using a pH meter: micro-pH 200 (Crison Instruments S.A., Barcelona, Spain).

The morphological examination of the PCE-NF was conducted using a Jeol JEM 1010 transmission electron microscope (TEM) (Jeol, Tokyo, Japan). Negative uranyl acetate staining was performed using 5 µL of the undiluted formula and 20 µL of 2% uranyl acetate. The sample was placed on UV-activated carbon-coated copper grids, which were then air-dried for 24 h before TEM observation.

The entrapment efficiency (EE) of luteolin 7-(6″-acetylglucoside) in PCE-NF was determined by ultrafiltration using Amicon^®^ Ultra centrifugal filter units (molecular weight cut-off: 10 kDa). Briefly, 1 mL of the freshly prepared nanoemulsion was placed in the filter unit and centrifuged at 4000× *g* for 30 min at 25 °C. The filtrate, containing the unencapsulated (free) drug, was collected. In parallel, the total drug content was determined by diluting and disrupting an aliquot of PCE-NF in the mobile phase, composed of acidified water (5% glacial acetic acid) and acetonitrile. Both samples were analyzed by HPLC-DAD under the previously validated conditions (Section 2.3). The entrapment efficiency (EE%) was calculated using Equation (1):

(1) $$\mathrm{EE}\%\;=\;\frac{C_{\mathrm{total}}\;-\;C_{\mathrm{free}}}{C_{\mathrm{total}}}\times 100$$

where C_total_ is the total drug content in PCE-NF and C_free_ is the amount of unencapsulated drug quantified in the filtrate.

Rheological measurements were performed with a Haake Rheostress^®^ 1 rheometer (Thermo Fisher Scientific, Karlsruhe, Germany) linked to a thermostatic circulator, Thermo Haake Phoenix II + Haake C25P, and a computer Pc equipped with Haake RheoWin^®^ Job Manager and Data Manager software v. 4.91. Steady-state measurements were conducted using a cone-and-plate geometry (C60/2°Ti: 60 mm diameter, 2° angle) to determine the shear stress (τ) as a function of the shear rate (γ). Viscosity curves (η = f(γ)) and flow curves (τ = f(γ)) were obtained at 25 ± 0.1 °C. The shear rate ramp program included a 3 min ramp-up period from 0 to 100 s^−1^, a 1 min constant shear rate period at 100 s^−1^, and a 3 min ramp-down from 100 to 0 s^−1^. Data from the flow curves were fitted using mathematical models to identify the model that best matched the experimental rheological data observed. The correlation coefficient value (r) and chi-square value were used to evaluate the adequacy of the rheological profiles to the mathematical models. Steady-state viscosity (η, mPa·s) was determined from the constant shear section at 100 s^−1^.

For the extensibility evaluation, PCE-NF and Blank-NF (0.3 mL) were placed on a steel plate circle and covered with a glass plate. Different weights were placed above the glass plate for 1 min each. The increasing diameter (cm) of the formulations was noted each time, and a curve of the increased surface area (cm^2^) was plotted as a function of increasing weight. The extensibility was fitted to the Boltzmann Sigmoidal model described in Equation (2):

(2) $$Y=\;\frac{Bottom\;+\;(\;Top-Bottom)}{1+\exp\left(\frac{V50-X}{Slope}\right)}$$

### 2.6. Stability Study

The physical stability of the nanostructured formulations was assessed using an optical analyzer, Turbiscan^®^ Lab (Formulation, l’Union, France), with samples (20 mL) placed in a glass measurement cell. This method allows the detection of the destabilization phenomena of nanoformulations by multiple light scattering analysis of backscattering and transmission profiles using pulsed near-infrared light (λ  =  880 nm) on day 0 and after storge at 4 °C for 30 days. A deviation of ≤2% in the backscattering profiles indicates no significant changes in particle size, while deviations exceeding ±10% suggest instability in the formulations.

### 2.7. In Vitro Release Assay

The released amount of luteolin-acetylglucoside from PCE-NF was assessed using vertical diffusion Franz cells (FDC-400, Vidra-Foc, Barcelona, Spain). A dialysis membrane (12 kDa, Dialysis Tubing Visking, Medicell International Ltd., London, UK) priorly hydrated was placed between the donor and receptor compartments. The effective diffusional area was 0.64 cm^2^. The receptor compartment was filled with Milli-Q water, where sink conditions were maintained, and 0.1 mL of the formulations was added to the donor compartment. Samples (0.2 mL) were collected at a predetermined time and replaced with the same amount of receptor phase medium. The samples underwent an HPLC-DAD analysis which was previously validated to quantify the luteolin-acetylglucoside [14]. The released amount of luteolin-acetylglucoside, expressed as mean ± SD (*n* = 3), was plotted versus time (h) using GraphPad^®^. The experimental release data were fitted to five kinetic models: the first-order, hyperbolic, Higuchi, Weibull, and Korsmeyer–Peppas models. The goodness-of-fit was evaluated based on the correlation coefficient (*r*^2^), and the model with the highest *r*^2^ value was selected as the most representative of the release profile.

### 2.8. Ex Vivo Skin Permeation

Human skin samples measuring 0.5 µm in thickness were obtained during an abdominal lipectomy performed on a healthy 38-year-old woman (Hospital of Barcelona, SCIAS, Barcelona, Spain) in accordance with the Hospital of Barcelona’s Ethical Committee (number 001, dated 20 January 2016). Some skin samples were subjected to microneedles rolling across the skin surface to mimic damaged skin. Healthy and injured skin samples were placed between the donor/receptor compartments of the vertical diffusion Franz cells (FDC-400, Vidra-Foc, Barcelona, Spain) with the *stratum corneum* facing up. The integrity of these 0.4 mm thick skin samples was evaluated by transepidermal water loss (TEWL) using Tewameter TM 300 (Courage & Khazaka Electronics GmbH; Cologne, Germany), and those with results below 10 g/m^2^h were used. The receptor compartment was filled with Milli-Q water, which was kept at 32 °C and under stirring at 600 rpm to guarantee sink conditions. PCE-NF (0.1 mL) was added to the donor compartment, and 0.2 mL aliquots were collected at a predetermined time and replaced with the same amount of receptor phase medium. The luteolin-acetylglucoside was quantified by HPLC-DAD analysis (Section 2.3).

The permeation parameters were derived by plotting the cumulative amount of luteolin-acetylglucoside permeated through the skin as a function of time (h) and determining the plot intercept and slope through regression analysis using GraphPad^®^ for the calculation of the steady-state flux (*J*). The permeability coefficient (*Kp*, cm/h) was determined by dividing the flux (*J*) by the initial extract concentration added to the donor compartment (C0) according to Equation (3):

(3) $$Kp=\frac{J}{C0}$$

At the end of the permeation study, the skin was removed and washed with sodium lauryl sulfate (0.05%) and distilled water. The permeation area was cut and weighed. The amount of luteolin-acetylglucoside retained in the skin (µg/g skin/cm^2^) was extracted with Milli-Q water using a sonication method for 50 min in an ultrasound bath and then determined through HPLC analysis.

### 2.9. In Vitro Efficacy Study on Skin Cells

#### 2.9.1. Cell Viability Study

The biocompatibility of the PCE-NF was assessed through the MTT cell viability assay. Briefly, 3T3-L1 (fibroblasts) and HaCaT (keratinocytes) cells were seeded in 96-well plates and incubated overnight. The cells were then treated with different concentrations (12.5, 6.25, 3.125, 1.56 and 0.78 µg/mL) of either the free extract of *P. crinita*, PCE-NF or Blank-NF and incubated for 48 h. All samples were diluted in DMEM (Dulbecco’s modified Eagle’s medium). MTT solution (50 µL, 1 mg/mL) was added to each well after incubation, and then incubated for 2–4 h at 37 °C with 5% CO_2_. The formazan crystals formed, were dissolved with DMSO (100 µL), and the absorbance was measured using a microplate reader (ThermoScientific (Vantaa, Finland)) at 570 nm.

#### 2.9.2. Scratch Assay

The 3T3-L1 (5 × 10^5^ cells/well) and HaCaT (10^6^ cells/well) cells were seeded in 6-well plates and incubated for 24 h at 37 °C with 5% CO_2_. After reaching confluence, the cell layers were scratched using a sterile tip and treated with two concentrations (6.25 and 3.25 µg/mL) of either the free extract of *P. crinita*, PCE-NF or Blank-NF. Images were taken of different wound areas using an inverted DM-IRBE microscope (Leica, Rueil-Malmaison, France), after 16 and 24 h for 3T3-L1 fibroblasts, and after 16, 24 and 36 h for keratinocytes. Data were analyzed using ImageJ software (ImageJ v 1.54), and the wound closure percentage was calculated in comparison to that under the condition with control cells.

### 2.10. Tolerance Studies

#### 2.10.1. In Vitro HET-CAM Assay

The Hen’s Egg Test Chorioallantoic Membrane was performed to assess the risk of irritation of NF on 10-day-old chicken eggs (supplied by the G.A.L.L.S.A. farm, Tarragona, Spain). The eggshell and inner membrane were removed to reveal the chorioallantoic membrane (CAM) dividing the embryo from the air chamber. PCE-NF and Blank-NF (0.3 mL) were applied on the CAM along with a negative control (0.9% NaCl) and a positive control (0.1 N solution of NaOH). The eggs were monitored for 5 min to detect any irritation reactions (hemorrhage, lysis, and/or coagulation). The irritation index or irritation score (IS) was then calculated using Equation (4):

(4) $$IS=\left[\frac{\left(301-hemorrhage\;time\right)}{300}\times 5\right]+\left[\frac{\left(301-lysis\;time\right)}{300}\times 7\right]+\left[\frac{\left(301-coagulation\;time\right)}{300}\times 9\right]$$

The classification used was as follows: IS between 0 and 0.99: non-irritant; IS between 1.0 and 4.99: slightly irritant; IS between 5.0 and 9.99: moderately irritant; and IS between 10.0 and 21.0: extremely irritant.

#### 2.10.2. In Vivo Tolerance Study

A tolerance study was conducted through the measurement of two parameters: transepidermal water loss (TEWL), which is a measure used to evaluate the retrograde water permeation through the skin indicating the overall amount of water vapor released, and *stratum corneum* hydration (SCH), which reveals the capacitance fluctuation of the dielectric properties of the *stratum corneum* caused by skin hydration variations. The TEWL was measured using Tewameter^®^ TM 300 (Courage-Khazaka electronic GmbH, Cologne, Germany), and the SCH was measured using Corneometer^®^ CM 825 (Courage-Khazaka electronic GmbH, Cologne, Germany). Measurements were taken at different time intervals including 15 and 120 min after treatment application on the left forearm in comparison to the basal level (at T = 0, before applying the treatments).

### 2.11. Statistical Analysis

Statistical significance was determined using GraphPad Prism 8.0.2 (263) (GraphPad Software, San Diego, CA, USA). The Tukey post hoc test was performed following two-way ANOVA for multi-group comparison. Student’s *t*-test was conducted for two-group comparison. Values of *p* < 0.05 were considered statistically significant.

## 3. Results

### 3.1. Physicochemical Characterization

**Figure 1:**
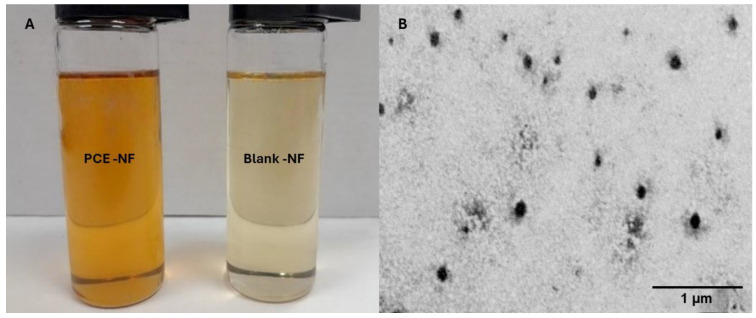
Physical characterization of *P. crinita* extract-loaded nanoemulsion (PCE-NF). (**A**) Physical appearance of PCE-NF and Blank-NF and (**B**) transmission electron microscopy (TEM) image of PCE-NF (magnification 20,000×).

The final formulation of PCE-NF was composed of 0.5% of *P. crinita* extract, 14.88% of Labrafac lipophile^®^ as the oil phase, 53.48% of Labrasol^®^ as a surfactant, 13.28% of Plurol oleique^®^ as a co-surfactant, and 17.88% of propylene glycol as the hydrophilic phase. PCE-NF, composed of Labrafac lipophile^®^, Labrasol^®^, Plurol^®^ oleique, and propylene glycol, showed a slightly yellowish color and homogenous appearance, with no sign of drug precipitation (Figure 1A). This formulation showed an adequate pH value for the topical administration of 6.34 ± 0.01. PCE-NF presented a mean droplet size of 212.27 ± 26.50 nm with a PdI of 0.36, while Blank-NF had a smaller droplet size (79.55 ± 2.71 nm) with a PdI of 0.32. The droplet size revealed by TEM was consistent with those obtained by photon correlation spectroscopy (Figure 1B). The content of luteolin-acetylglucoside in the *Phlomis crinita* extract was determined by HPLC-DAD and was found to be 168.5 mg/g of extract. The quantification of this compound in PCE-NF revealed a concentration of 0.83 mg/g of the formulation, indicating the successful incorporation of the active component. The EE% of luteolin-acetylglucoside in the nanoemulsion was determined by ultrafiltration and calculated to be 93.58 ± 0.25%, confirming the high retention of the compound within the formulation.

**Figure 2:**
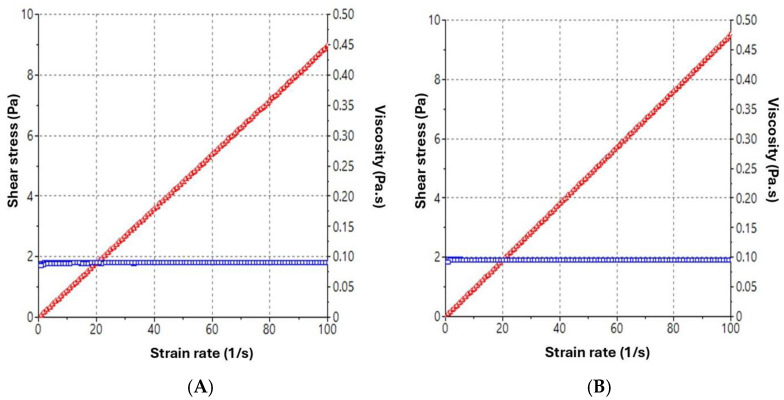
Rheological profiles indicating the viscosity curve (blue line) and the flow curve (red line). (**A**) Blank-NF and (**B**) *P. crinita* extract-loaded nanoemulsion (PCE-NF).

Steady-state rheological measurements as a function of shear rate are presented in Figure 2. PCE-NF and Blank-NF displayed typical Newtonian profiles, characterized by a linear relationship between shear stress and shear rate (flow curve) with no thixotropy, whereas the viscosity remained constant. The viscosity values at 100 s^−1^ were 89.78 ± 0.09 and 95.45 ± 0.08 mPa·s for Blank-NF and PCE-NF, respectively.

**Figure 3:**
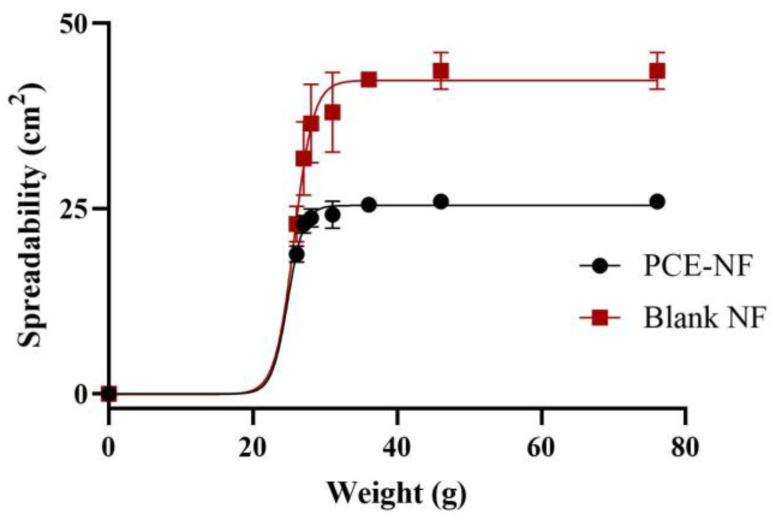
Spreadability profiles of *P. crinita* extract-loaded nanoemulsion (PCE-NF) and Blank-NF. Data are presented as the mean ± SD, (*n* = 3).

The spreading properties of both formulations are shown in Figure 3; it can be observed that the blank formulation exhibited higher extensibility values compared to the formulation containing the *P. crinita* extract (25.62 ± 0.24 and 43.27 ± 0.64 cm^2^ for PCE-NF and Blank-NF, respectively). The Boltzmann sigmoidal model was the model with the best adjustment quality (r^2^ > 0.99).

### 3.2. Stability Studies

**Figure 4:**
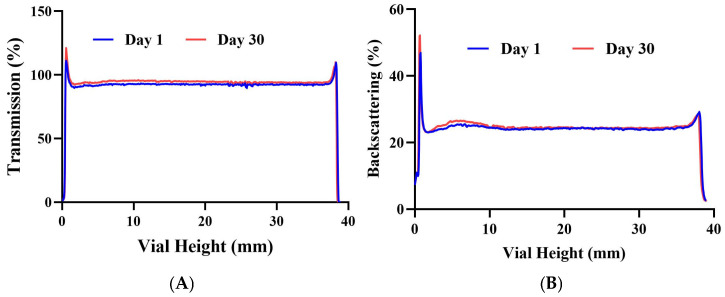
Physical stability assessment of *P. crinita* extract-loaded nanoemulsion (PCE-NF) on day 1 and day 30 of storage at 4 °C. (**A**) Transmission profile and (**B**) backscattering profile.

The transmission profile (%) of PCE-NF over 30 days is illustrated in Figure 4. The peaks on both the left and right sides of the curve are attributed to the meniscus formed at the interface between the formulation and the glass. Throughout the 30 days of storage at 4 °C, this formulation demonstrated physical stability, showing no signs of precipitation or any changes in the system. In the same way, the backscattering profile demonstrated variations of less than ±10% at 4 °C after 30 days of preparation, suggesting that the formulation was stable.

### 3.3. In Vitro Release Assay

**Figure 5:**
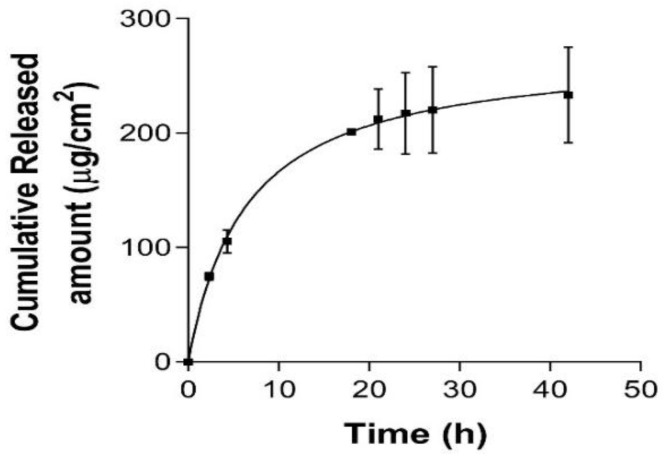
Luteolin-acetylglucoside release profile from *P. crinita* extract-loaded nanoemulsion (PCE-NF). Data are presented as the mean ± SD, (*n* = 5).

As shown in Figure 5, the release profile of luteolin-acetylglucoside from the nanoformulation was best fitted to the one-site binding hyperbola model (r^2^ > 0.9). These results demonstrated a burst release of luteolin-acetylglucoside during the first hours followed by sustained release up to 42 h, with a B_max_ of 272.7 µg and a release constant (K_d_) of 6.388 h.

### 3.4. Ex Vivo Skin Permeation

The permeation parameters of luteolin-acetylglucoside through healthy and injured skin revealed a significant increase by 1.3-fold in the permeation flux and permeability coefficient values on injured compared to healthy skin. However, no significant difference was observed in the amount of luteolin-acetylglucoside permeated after 27 h, as depicted in Table 1.

**Figure 6:**
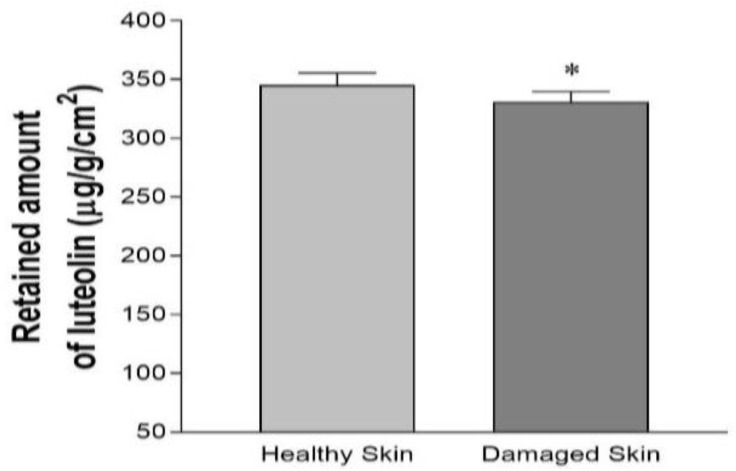
Retained amount of luteolin-acetylglucoside in healthy and damaged skin. Data are expressed as the mean ± SD (*n* = 5). * *p* < 0.05.

Figure 6 demonstrates the retention results of luteolin-acetylglucoside in the skin. Healthy skin exhibited a significantly higher retained amount of the drug compared to injured skin (*p* < 0.05).

### 3.5. In Vitro Study on Skin Cells

#### 3.5.1. Cell Viability

**Figure 7:**
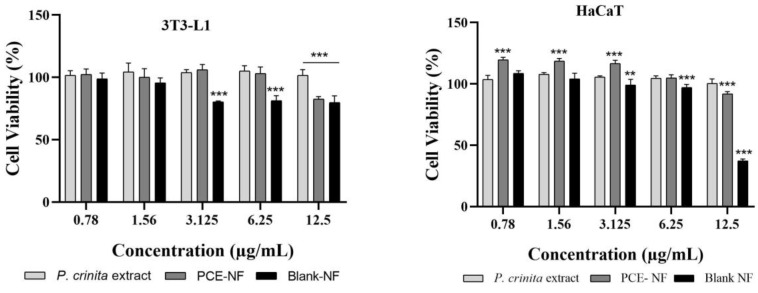
Cell viability percentage of 3T3-L1 and HaCaT cells after 48 h of treatment with the free *P. crinita* extract, the *P. crinita* extract-loaded nanoemulsion (PCE-NF) and Blank-NF. Data are expressed as the mean ± SD (*n* = 5). ** *p* < 0.01 and *** *p* < 0.001.

The MTT results (Figure 7) show that the free extract of *P. crinita* and PCE-NF had no cytotoxic effect on both cell lines. Moreover, PCE-NF revealed significantly higher viability than the *P. crinita* extract in HaCaT cells. Moreover, HaCaT cells showed a viability greater than 80% after the exposition of Blank-NF up to a concentration of 6.25 µg/mL, being non-cytotoxic according to the ISO standard [20,21].

#### 3.5.2. Wound Healing Properties Using the Scratch Assay

**Figure 8:**
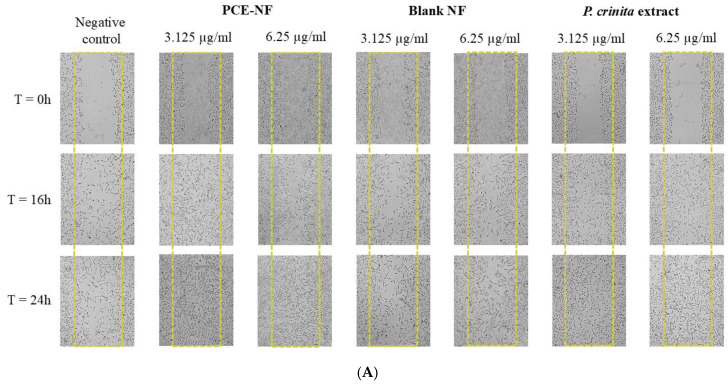

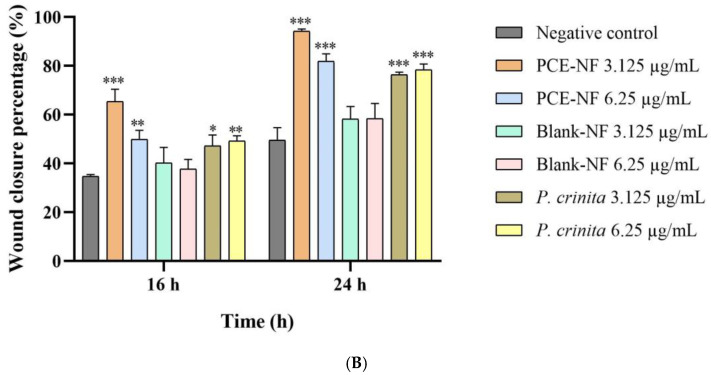
In vitro wound healing studies. (**A**) Representative microscopic images of 3T3-L1 fibroblast scratch assay using treatments with *P. crinita* extract-loaded nanoemulsion (PCE-NF), Blank-NF and free *P. crinita* extract at 3.125 and 6.25 µg/mL, at 0, 16 and 24 h. (**B**) Percentage of wound closure measured from microscopic images. Data are presented as mean ± SD, (*n* = 3). Significant statistical differences: * *p* < 0.05; ** *p* < 0.01 and *** *p* < 0.001.

To assess the effect of *P. crinita* extract and PCE-NF on cell migration using the scratch assay, the concentrations 3.125 and 6.25 µg/mL, which showed no toxicity to both cells, were selected based on the results of the cell viability assay. These results revealed that, after 16 h of incubation, the 3T3-L1 cells exhibited a significant increase in wound closure, with a value of 65.5 ± 5%, using PCE-NF at 3.125 µg/mL, and after 24 h of incubation, the wound closure percentage reached a value of 94.24 ± 0.9%, which is significantly higher than that of the free extract at both concentrations (76.33 ± 1.16% and 78.5 ± 2.3% for *P. crinita* extract at 3.125 and 6.25 µg/mL, respectively) (Figure 8).

**Figure 9:**
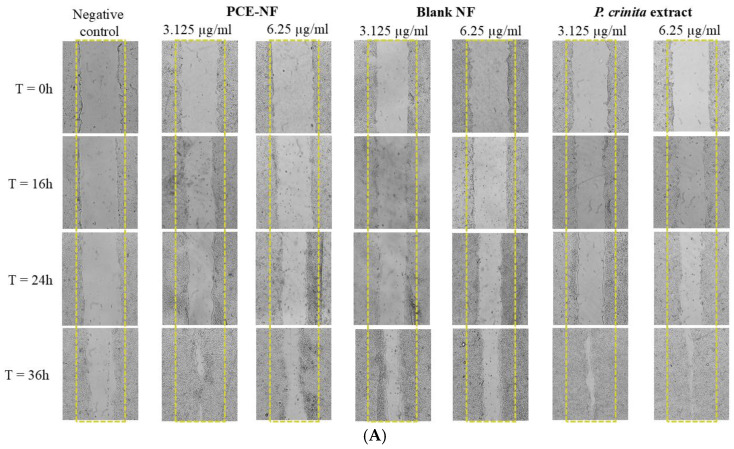

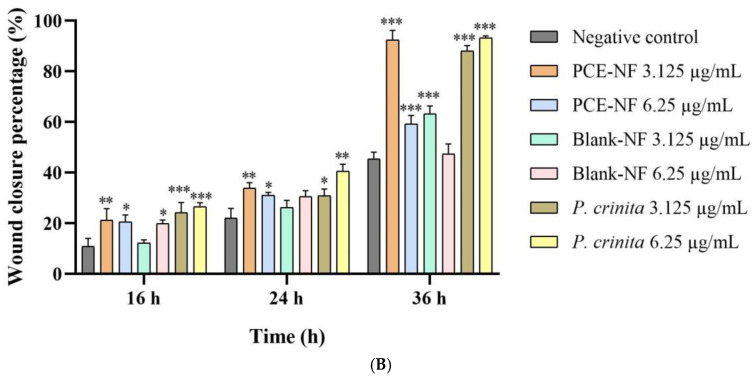
In vitro wound healing studies. (**A**) Representative microscopic images of HaCaT keratinocyte scratch assay using treatments with *P. crinita* extract-loaded nanoemulsion (PCE-NF), Blank-NF and free *P. crinita* extract at 3.125 and 6.25 µg/mL, at 0, 16, 24, and 36 h. (**B**) Percentage of wound closure measured from microscopic images. Data are presented as mean ± SD, (*n* = 3). Significant statistical differences: * *p* < 0.05; ** *p* < 0.01 and *** *p* < 0.001.

Furthermore, HaCaT cells treated with 3.125 µg/mL of PCE-NF showed a significant increase in wound closure after 36 h of incubation (92.4 ± 3.8%). This increase is almost equivalent to that observed with HaCaT cells treated with 6.25 µg/mL of free extract (93.3 ± 0.73%) (Figure 9).

### 3.6. Tolerance Studies

#### 3.6.1. In Vitro HET-CAM Assay

**Figure 10:**
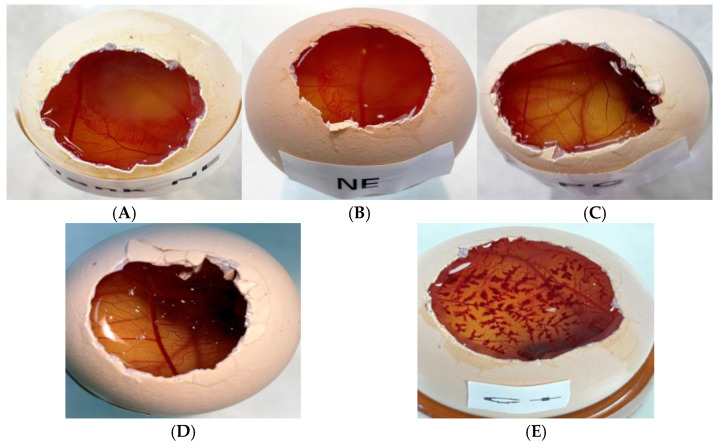
Assessment of the formulations’ irritant effects using HET-CAM: (**A**) Blank- NF; (**B**) PCE-NF; (**C**) *P. crinita* extract; (**D**) negative control (saline solution); and (**E**) positive control (0.1 N sodium hydroxide solution).

To determine the possible rapid irritation reaction, the HET-CAM test was conducted (Figure 10). The irritation score of the positive control was 11.97 ± 0.62, which is classified as extremely irritant, while the negative control revealed no signs of irritation within 5 min. The free *P. crinita* extract also had no irritating signs such as hemorrhage, lysis or coagulation, thus behaving like the negative control. However, Blank-NF and PCE-NF appeared to be slightly irritants as the irritation scores were 3.01 ± 0.16 and 2.07 ± 0.40, respectively.

#### 3.6.2. In Vivo Tolerance Study

**Figure 11:**
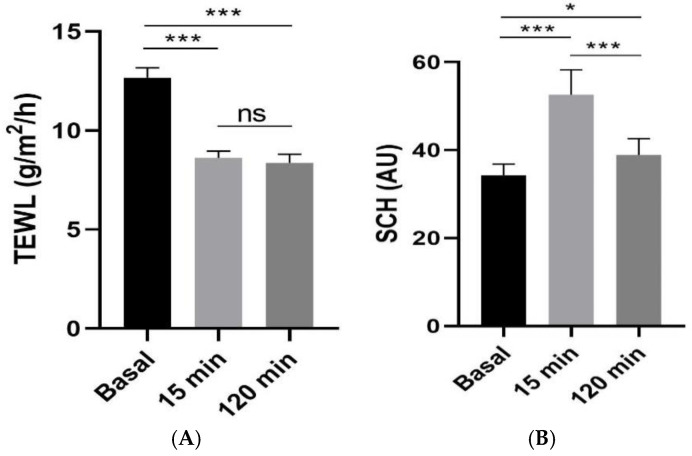
Biomechanical properties in human volunteers before application (basal), 15 min and 120 min post-application of Blan-NF. (**A**) TEWL: transepidermal water loss; (**B**) SCH: *Stratum corneum* hydration. Significant statistical differences: * *p* < 0.05; *** *p* < 0.001, ns = non-significant.

The results revealed that the TEWL values significantly decreased after 15 min of the application of Blank-NF whereas the hydration levels of the *stratum corneum* increased significantly with a tendency to return to the basal state (Figure 11).

## 4. Discussion

This work presents a nanoemulsion-based formulation loaded with *P. crinita* extract, specifically designed to enhance the topical delivery and skin permeation of luteolin-acetylglucoside, its main active compound. While previous studies have incorporated this extract into polymeric nanoparticles, nanoemulsions offer several advantages, including greater physical stability, easier scalability, and superior sensory properties for topical use [14]. In particular, the presence of surfactants and co-surfactants in the formulation plays a critical role in disrupting the *stratum corneum* lipid structure, thereby improving skin adherence, enhancing permeation, and increasing the bioavailability of poorly water-soluble compounds [24]. As a result, nanoemulsions have gained growing attention in wound therapy research and are considered a highly effective delivery platform for both synthetic and natural active ingredients [21]. The *P. crinita*-loaded nanoemulsion (PCE-NF) obtained in this study showed a homogenous and transparent appearance, with no signs of precipitation or phase separation, and pH values suitable for skin application. When developing novel formulations, the average particle size and particle size distribution are essential factors to take into consideration since low values of these parameters indicate an increase in the product’s physical stability and shelf life [25]. In this study, PCE-NF had moderate size distribution of droplets with a spherical morphology, making it suitable for topical application [26]. The incorporation of *P. crinita* extract in the nanoformulation increased the droplet size (212.27 ± 26.50 nm), as expected, with respect to the Blank-NF (79.55 ± 2.71 nm) [23,27,28]. The high entrapment efficiency observed (93.58 ± 0.25%) reflects the strong affinity of luteolin acetylglucoside for the lipophilic core of the nanoemulsion, likely facilitated by its physicochemical properties and the solubilizing capacity of the surfactant system. This efficient incorporation is advantageous for ensuring sustained release and localized delivery at the application site. Rheological parameters influence sensory characteristics, the accuracy of filling and dosing processes, and formulation spreadability, and can significantly influence biopharmaceutical performance, including the drug release rate. PCE-NF exhibited Newtonian behavior, which, while common in nanoemulsions, may limit residence time under dynamic conditions such as mucosal movement or application on vertical skin surfaces, unlike pseudoplastic or thixotropic systems, which tend to increase residence time through shear-dependent viscosity changes [29]. The slight increase in viscosity observed in PCE-NF (95.45 ± 0.08 mPa·s) compared to that in Blank-NF (89.78 ± 0.09 mPa·s) suggests that the addition of the extract slightly thickens the formulation, which could enhance its skin adherence. Nonetheless, to improve adherence and prolong residence time in future applications, strategies such as the incorporation of mucoadhesive polymers or viscosity enhancers could be explored to impart shear-thinning or viscoelastic properties. Spreadability testing is a key aspect in the development of dermatological and pharmaceutical products, as it directly influences efficacy, patient comfort, and treatment adherence. Good spreadability can also improve topical bioavailability [30]. In this study, PCE-NF and Blank-NF spread easily under moderate pressure, with Blank-NF displaying slightly higher spreadability. These results suggest that PCE-NF could be delivered in aerosol form, minimizing direct contact with the skin, an advantage when treating large or irregular areas. This facilitates uniform dosing of the active ingredient and enhances patient compliance [31].

The stability of drug-loaded nanosystems is challenging during pharmaceutical drug development, which could lead to the formation of aggregated particles with a loss of nanoscale properties [23,32]. The physical stability study of the nanostructured formulation was carried out using two synchronized detectors. First, the backscattering detector measured the light backscattered by the droplets, allowing for the analysis of particle size changes. Our findings demonstrated good short-term stability, indicating no variation in particle size throughout the period investigated. Secondly, the transmission detector measured the light flux transmitted through the formulation to analyze particle migration [24]. The transmission profiles confirmed the physical stability of the formulation since no signs of sedimentation, flocculation, creaming or coalescence were observed. The high physical stability observed for PCE-NF can be attributed to the steric stabilization provided by the non-ionic surfactants (Labrasol^®^ and Plurol^®^ Oleique) present in the formulation. These surfactants form a protective layer around the oil droplets, preventing their coalescence by creating a physical barrier rather than relying on electrostatic repulsion [33]. While the short-term stability results are promising, for clinical use, these findings should be confirmed through accelerated and long-term stability studies under controlled temperature and humidity conditions, as established by ICH guidelines [34].

The drug’s rate and release profile from the vehicle represents valuable information as it can serve as quality control data to predict in vivo behavior and explore the underlying mechanisms [35,36]. Moreover, release and permeation studies are important for estimating drug bioavailability in dermal formulations, helping to evaluate a drug’s effectiveness [37]. The results of this study demonstrated a slow and sustained release of luteolin-acetylglucoside from the prepared nanosystem following a hyperbola kinetic model with a K_d_ of 6.388 h. These findings suggest that the extract’s incorporation in a nanosystem may improve its bioavailability and prolong its therapeutic activity at wound sites, which in turn could enhance its wound healing potential through continuous bioactive delivery.

Previous studies have shown that nanoemulsions function as carriers that improve penetration and permeation into the skin by overcoming the *stratum corneum* barrier [26]. The ex vivo permeation study was performed using healthy and injured skin to mimic skin barrier disorders such as wounds and cuts. Our findings revealed that the permeation of luteolin-acetylglucoside was higher and faster in the injured than healthy skin (Table 1), showing higher values for the flux (*J*), permeability coefficient (*Kp*) and permeated amount at 27 h (Q_27h_). These results indicate that the external hydrophobic layer of the skin was altered, facilitating a faster and easier penetration of substances through the skin barrier [38]. However, the retained amount of luteolin-acetylglucoside (Q_ret_) within the skin was higher for healthy skin (345.02 µg/g/cm^2^) compared to injured skin (330.88 µg/g/cm^2^), which suggests that the *stratum corneum* acts as a reservoir that favors its local and prolonged effect. The enhanced penetration of the luteolin-acetylglucoside through the skin could be due to the formulation components, since previous reports have shown that Plurol^®^ Oleique enhances skin permeation through disturbing the skin lipid arrangement [39]. Moreover, Labrasol^®^ has been shown to modulate epidermal tight junctions leading to penetration enhancement. Previous studies indicated that the disruption of the *stratum corneum* increased the penetration flux of nanoparticles, resulting in a higher amount within the deeper layers of the epidermis and dermis [38]. These results are promising for wound healing, as the formulation enables the delivery and retention of the extract to deeper layers whether the skin is damaged or intact.

The scratch assay is one of the methods that has been shown to be a useful and affordable tool for gaining preliminary insights into how plant preparations or their identified components might positively impact the development of new tissue [4,40]. Polymeric nanoparticles of *P. crinita* extract have demonstrated its potential use as a wound healing agent through the promotion of fibroblast migration and proliferation [14]. In this study, we assessed the migratory and proliferative effects on two types of skin cells, fibroblasts and keratinocytes, which are pivotal to the epithelialization process of wound healing. Significant wound closure was observed with the free extract of *P. crinita* at both tested concentrations. The higher concentration resulted in an increased closure percentage, indicating a dose–response relationship. Remarkably, the efficacy of PCE-NF at a concentration of 3.125 µg/mL was of 94.24 ± 0.9% and 92.4 ± 3.8% in 3T3-L1 and HaCaT cells, respectively. These percentages of wound closure were higher than those observed in the free extract at 6.25 µg/mL, suggesting that the delivery nanosystem may enhance wound healing, therefore being potentially more effective.

The HET-CAM test is a simple, sensitive, and a valid alternative that has been used to determine whether a drug formulation could cause ocular irritation [41]. Despite being intended to evaluate the potential of ocular irritation, this assay was for cases where the formulation came into contact with the ocular area, either accidentally or due to its proximity to the treated area [14,41]. Our findings revealed that the level of eye irritation of Blank-NF and PCE-NF is significantly lower compared to that of the positive control. The safety of the excipients used in PCE-NF, particularly Labrasol^®^ and Plurol^®^ oleique, is supported by their long-standing use in topical pharmaceutical and cosmetic products. Both excipients have been widely employed as solubilizers and co-surfactants due to their favorable properties and good skin compatibility [42]. Although a slight irritant effect was observed in the HET-CAM assay, this finding is consistent with previous reports and does not compromise their suitability for dermal application, especially at the concentrations used, but it is necessary to recommend avoiding the formulation coming into contact with the eyes [43,44]. On the other hand, the viability studies in skin cells demonstrated that PCE-NF and free *P. crinita* extract do not have cytotoxic effects on both cell types (HaCaT and 3T3-L1 cells). The high cell viability values, some exceeding 100%, reflect the absence of cytotoxic effects, allowing cells to grow under normal conditions with slight variations in metabolic activity or proliferation. This can occur due to a mild stimulatory effect on cell growth beyond that of untreated controls, which is consistent with the wound healing potential of the formulation [3]. In either case, these values confirm that PCE-NE did not negatively affect cell viability.

The biomechanical parameters’ evolution before and after the application of Blank-NF was studied to assess its impact on skin hydration and integrity. The results of this study showed a decrease in TEWL and increase in SCH measurements, suggesting that this formulation benefits skin barrier function and hydration levels simultaneously. Reduced TEWL indicates that the formulation is preserving skin moisture levels, preventing water loss, and strengthening the skin barrier. On the other hand, an increase in SCH suggests that the formulation is effectively hydrating the skin and improving its moisture retention capacity [36].

The above-mentioned findings suggest that the integration of bioactive natural compounds into nanosystems represents a promising strategy for advancing wound healing therapies. This approach not only addresses the limitations of conventional treatments, such as the poor solubility, rapid degradation, and low bioavailability of natural agents, but also enables controlled and sustained release at the wound site, enhancing therapeutic efficacy and safety. Furthermore, the potential for combination therapies, such as the co-delivery of phytocompounds with antibiotics within nanocarriers, might offer synergistic effects that can accelerate tissue regeneration and improve antimicrobial action.

## 5. Conclusions

*Phlomis crinita* extract was successfully incorporated into a physically stable nanostructured formulation (PCE-NF) which demonstrated favorable physicochemical properties, high stability, biocompatibility, and non-cytotoxic behavior. Notably, PCE-NF facilitated the sustained release of its major active compound, luteolin-acetylglucoside, and exhibited improved skin permeation and retention. This formulation showed notable efficacy in promoting wound closure, highlighting its potential to enhance cell migration in both fibroblasts and keratinocytes. The integration of a natural product into a nanoemulsion delivery system represents a significant advancement in the design of topical treatments. By addressing the challenges of drug stability, skin penetration, and controlled release, this work supports the future development of plant-based, nanostructured therapies for skin regeneration and wound management.

## Figures and Tables

**Table 1:** Permeation parameters of *P. crinita* extract-loaded nanoemulsion (PCE-NF).

Biopharmaceutical Parameters	Formulation NF	
	Healthy Skin	Injured Skin
*J* (µg/h/cm^2^)	0.369 ± 0.07	0.480 ± 0.005 **
*K*_*p*_ × 10^−5^ (cm/h)	7.4 ± 0.8	9.6 ± 0.07 ***
Q_27h_ (µg)	22.01 ± 1.26	21.44 ± 1.7

Flux (*J*), permeability coefficient (*Kp*), and permeated amount of luteolin-acetylglucoside at 27 h (Q_27h_). Values are reported as the mean ± SD (*n* = 5). Statistical difference: ** *p* < 0.01 and *** *p* < 0.001.

## Data Availability

The data presented in this study are available in this article.

## Supplementary Materials

The following supporting information can be downloaded at https://www.mdpi.com/article/10.3390/pharmaceutics17091093/s1: Figure S1: HPLC chromatogram at 330 nm: (A) blank nanoemulsion; (B) standard solution of luteolin 7-(6″-acetylglucoside) (25 µg/mL); (C) nanoemulsion formulation (PCE-NF).
