# Supplementary material for: Efficacy of Phlomis crinita Extract-Loaded Nanostructured Formulation in Accelerating Wound Healing
Source: Pharmaceutics. 2025 Aug 22;17(9):1093. doi: 10.3390/pharmaceutics17091093 (PMC12473550; doi:10.3390/pharmaceutics17091093)
Supplement: Supplementary file 1 [file pharmaceutics-17-01093-s001.zip › pharmaceutics-3727367-supplementary.pdf]

Supplementary material

A.

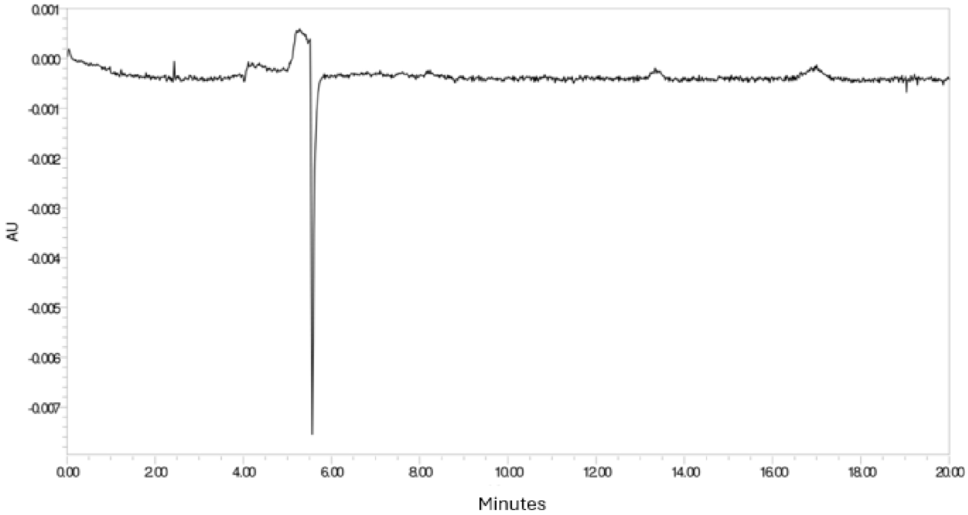

B.

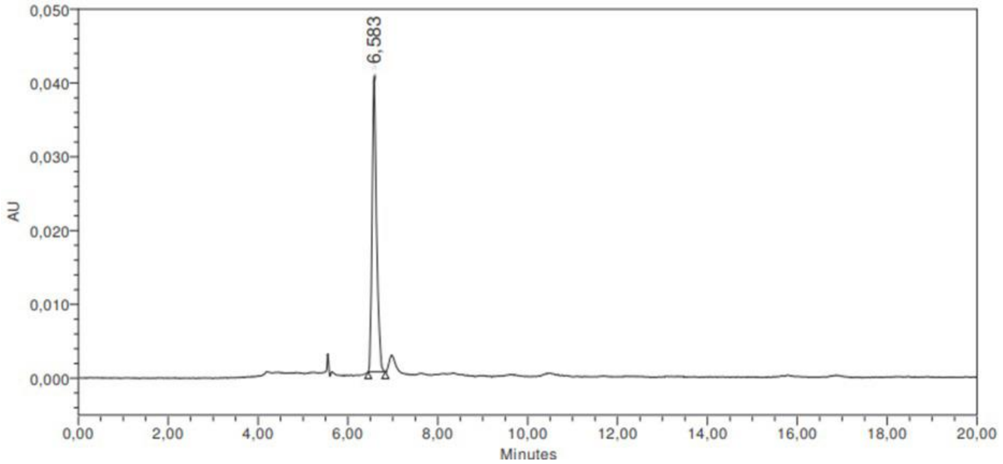

C.

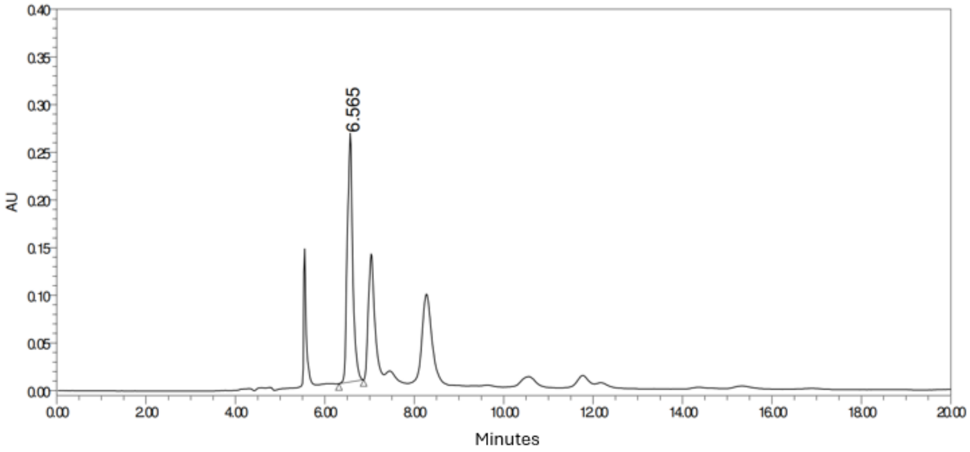

Figure S1. HPLC chromatogram at 330 nm. A. Blank nanoemulsion; B. Standard solution of luteolin 7-(6″-acetylglucoside) (25 µg/mL); C. Nanoemulsion formulation (PCE-NF).
